# Actinoporins: From the Structure and Function to the Generation of Biotechnological and Therapeutic Tools

**DOI:** 10.3390/biom10040539

**Published:** 2020-04-02

**Authors:** Santos Ramírez-Carreto, Beatriz Miranda-Zaragoza, Claudia Rodríguez-Almazán

**Affiliations:** Departamento de Medicina Molecular y Bioprocesos, Instituto de Biotecnología, Universidad Nacional Autónoma de México, Avenida Universidad 2001, Cuernavaca, Morelos 62210, Mexico; santos@ibt.unam.mx (S.R.-C.); bemiza@ibt.unam.mx (B.M.-Z.)

**Keywords:** Pore-forming toxin, actinoporin, immunotoxin, nanopore, sphingomyelin, vaccine design

## Abstract

Actinoporins (APs) are a family of pore-forming toxins (PFTs) from sea anemones. These biomolecules exhibit the ability to exist as soluble monomers within an aqueous medium or as constitutively open oligomers in biological membranes. Through their conformational plasticity, actinoporins are considered good candidate molecules to be included for the rational design of molecular tools, such as immunotoxins directed against tumor cells and stochastic biosensors based on nanopores to analyze unique DNA or protein molecules. Additionally, the ability of these proteins to bind to sphingomyelin (SM) facilitates their use for the design of molecular probes to identify SM in the cells. The immunomodulatory activity of actinoporins in liposomal formulations for vaccine development has also been evaluated. In this review, we describe the potential of actinoporins for use in the development of molecular tools that could be used for possible medical and biotechnological applications.

## 1. Introduction

Pore-forming toxins (PFTs) represent a group of structurally and functionally diverse molecules and possess the ability to adapt to the environment through conformational changes. They exist in a soluble conformation in an aqueous medium or form pores in the plasma membrane, thus allowing the passage of ions or molecules to produce an osmotic imbalance leading to cell lysis and death [1]. This intrinsic ability to produce cellular damage has been exploited for the development of drugs that can lead to tumor cell elimination [2,3,4,5]. Diverse bacterial PFTs have been used in the design of therapeutic molecules targeting cancer. One example involves immunotoxins, which are chimeric molecules that consist of a toxic fraction that is responsible for tumor cell damage and a fraction composed of an antibody that is responsible for directing the toxic fraction to a specific target [6,7,8].

Despite the potential exhibited by several bacterial PFTs for the design of immunotoxins, only one immunotoxin that is based on a region from the pore-forming protein of *Pseudomonas exotoxin* A has been approved by the FDA. This molecule is called moxetumomab pasudotox-tdfk (LUMOXITI^®^, AstraZeneca Pharmaceuticals LP), and it was approved in 2018 for application in B-cell cancer (NCT01829711;ClinicalTrials.gov) [9,10]. The immunogenicity and limited penetration capacity of some immunotoxins that were developed from bacterial toxins have highlighted the need to develop such molecular tools from PFTs from other sources, such as venoms [11]. The sea anemone venom contains some polypeptides with cytolytic and cytotoxic activity, among them enzymes, lectins, and PFTs [12,13,14]. Concerning sea anemones, three families of PFTs have been identified in their venom, namely the MACPF-like, aerolysin-like, and actinoporins [13,15], with the latter being the best characterized both functionally and structurally. 

Although the structural knowledge of the monomeric units and oligomeric forms of actinoporins has increased in recent years, their mechanism of action has not yet been fully described. These proteins were found to possess important physicochemical characteristics for the design of molecular tools with possible medical and biotechnological application [16,17,18,19]. Actinoporins (APs) bind to the membrane mainly through the specific recognition of lipids, mainly sphingomyelin (SM), and it has been demonstrated that the presence of cholesterol (Chol) promotes the formation of actinoporin pores in synthetic membranes [20,21,22]. Due to their specific interaction with SM, APs are ideal molecules for analyzing the lipid distribution and dynamics of SM in cell membranes [23,24,25], which may be especially relevant for the study of the membranes of certain cancer cells due to the presence of SM and Chol reported in these cells [26,27]. Based on this, actinoporins may have a higher activity towards tumor cells compared to non-tumor cell lines [14,28,29]. The cytotoxic activity of actinoporins in synergy with anticancer drugs has been tested in tumor cell lines, and it has been demonstrated to increase the effectiveness of certain anti-cancer drugs [28]. Based on these findings, actinoporins have been considered for use as components of immunotoxins against cancer cells [4,23,30,31,32]. 

PFTs have also demonstrated potential applicability for the design of stochastic sensors based on protein nanopores in a portable device used for sequencing of polypeptides and nucleic acids [33,34,35,36,37,38]. Actinoporins also possess characteristics that suggest they could prove useful for the design of biosensors that are based on nanopores and applied for the analysis of one unique molecule, polypeptide, or DNA [39,40,41,42]. These technologies could have an important impact in applications such as proteomics and the rapid diagnosis of diseases that are caused by intrinsically disordered proteins [41,42].

Two other possible applications of actinoporins that have been sparsely explored. First, as a component of the design of adjuvant tools for vaccine development, actinoporins could act as immunomodulators to enhance the specific cytotoxic cellular response of antigens within various liposomal formulations that could ultimately be used in the development of vaccines against intracellular pathogens or in cancer [43]. Additionally, because of their preference to bind to sphingomyelin, it has been proposed that actinoporins could be a great molecular tool in the analysis of sphingolipid distribution and dynamics in biological membranes [24]. 

Little is known about the detailed properties of actinoporins and their biotechnological applications. This manuscript is not intended to be a comprehensive review on actinoporins. Instead, it highlights the developments in actinoporin applications, in the biotechnological and biomedical fields. 

## 2. Structure and Function of Actinoporins

**Figure 1:**
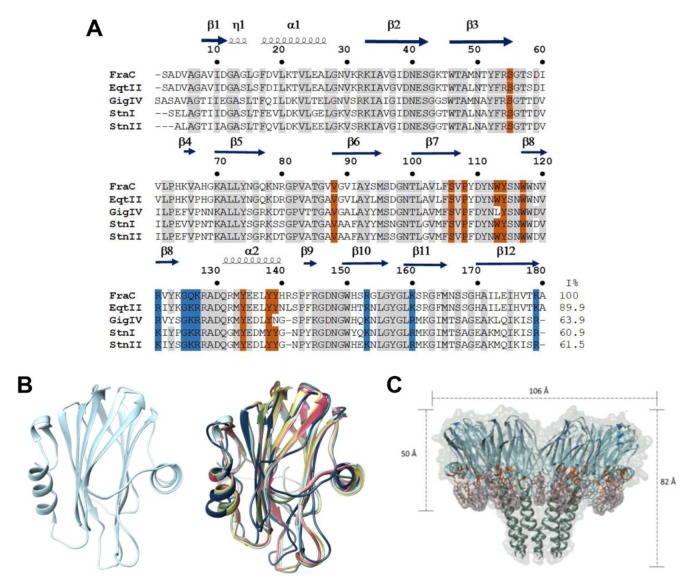
Structural characteristics of actinoporins. (**A**). Alignment of the most studied actinoporin sequences of the sea anemones *Actinia fragacea* (fragaceatoxin C, FraC), *Actinia equina* (equinatoxin II, EqtII), *Stichodactyla helianthus* (sticholysins I and II; StnI and StnII), *Stichodactyla gigantea* (gigantoxin-4, GigIV). The residues that interact with phosphocholine (POC) are highlighted in orange, the residues that interact with the membrane are in blue, and conserved residues are indicated in gray. (**B**). Structural alignment of actinoporins. All the Root mean square distances (RMSDs) were calculated using the structure of FraC as the reference structure, FraC (PDB 3VWI) in light blue, Stn I with a RMSD 1.208 Å (PDB 2KS4) in navy blue, Stn II with a RMSD 0.483 Å (PDB 1GWY) in pink, Eqt II with a RMSD 0.401 Å (PDB 1KD6) in yellow, GigIV with a RMSD 0.422 Å (I-TASSER model) in green [78]. (**C**). FraC oligomer (PDB 4TSY). FraC is shown in light blue, the residues that interact with POC are colorized in orange, and the residues that interact with the membrane are in blue. The zone in direct contact with the ligands is in purple, whilst the membrane is in green. The distances were obtained with the program UCSF Chimera^®^ [79].

Actinoporins (APs) are a multigene family of α-PFTs that are synthesized as a pre-pro-toxin that is enzymatically processed to generate a mature protein of ~170 -180 amino acids lacking Cys residues [44,45,46,47]. However, two actinoporin sequences with this residue present have been reported [48,49]. These proteins exhibit a characteristic molecular weight of ~20 kDa, typically possess a basic pI above 9, and display highly conserved primary and tertiary structures (Figure 1A,B). The most well studied actinoporins include equinatoxin II (EqtII) from *Actinia equina*, fragaceatoxin C (FraC) from *Actinia fragacea,* and sticholysin I and II (Stn I and Stn II) from *Stichodatyla helianthus* [50,51,52,53,54,55]. The tertiary structure of the monomeric soluble state consists of a single domain composed of a β-sandwich (two β-sheets each formed by five β-strands) each one connected by loops, where this core is flanked by two α-helices, one of these is located near the N-terminal region (Figure 1B) [56,57,58,59]. 

The actinoporins can alter the plasma membrane through the formation of pores, and this is strongly enhanced by the presence of SM [60]. This characteristic was first described by Bernheimer and Avigad more than 30 years ago, and SM was proposed as a specific receptor for actinoporins [61]. Studies in three actinoporins from *S. helianthus*, named sticholysin I (StnI) and II (StnII), and equinatoxin II (EqtII) from *A. equina*, corroborated the fact that SM has an important role in the recognition and permeabilization of plasma membranes by these toxins [62,63]. However, other studies proposed that actinoporins permeabilize liposomes that contain a mixture of Chol and phosphatidylcholine (PC). The cholesterol can induce microdomain formation, which leads to a membrane alteration for the accessibility of the toxins that interact with the phosphorylcholine group [28,64].

The presence of the N-terminal segment of actinoporins is essential for membrane permeabilization. By removing the N-terminal region of EqtII, the actinoporin is unable to lyse cells [65]. The flexibility of the N-terminal is necessary for the actinoporin to translocate to the lipid water interface. The addition of Cys residues to the N-terminal region of EqtII by mutagenesis results in disulfide bond which produces restrictions on the flexibility of the N-terminal and results in the toxin inactivation [66]. The actinoporin interaction with the membrane was confirmed by a structure analysis of EqtnII, StnI, and StnII in presence of phosphocholine (POC), identifying a cavity in the C-terminal region as lipid binding site [56,59,67].

The three-dimensional structure of StnII shows the existence of a POC binding site [55]. Thus, it was proposed, that the side chains of the residues Ser52, Val85, Ser103, Pro105, Tyr131, Tyr135, and Tyr 136 are located on the protein surface of StnII. The residues that make the POC binding site are conserved in the actinoporins sequenced to date [67]. In EqtII, Trp112 residue was identified in the vicinity of the POC binding site, and its participation as the most important residue in the initial interaction with the membrane was determined by recognizing SM [65,66]. The co-crystallization of StnII with POC, part of the polar head group of SM and PC, allowed the identification of a cluster of exposed amino acids (Ser52, Val85, Ser103, Pro105, Tyr111, Tyr131, Tyr135, and Tyr136) that interact with this molecule [55]. Molecular modeling and NMR analysis of EqtII, however, suggest that the side chain of Asp109 and Tyr113 and the main chains of Pro81 and Trp112 bind to SM by hydrogen bonds between the 2-NH and 3-OH groups of the SM ceramide. Experimental evidence suggests that these electrostatic interactions play a minor role, and the union is instead governed by hydrophobic interactions where aromatic amino acid residues and SM play a central role [68].

Based on the structure of EqtII, StnII, and FraC (Figure 1B) [56,68,69,70], the structural flexibility of actinoporins during transmembrane pore formation has been demonstrated. Actinoporin binds to the lipidic membrane by the specific recognition of SM by a region rich in aromatic residues, followed by the translocation of the N-terminal α-helix to the plasma membrane. Finally, oligomerization of three or four monomers occurs on the surface of the membrane, and the α-helices of the N-terminal are inserted into the membrane forming a pore, with a concomitant rearrangement of membrane lipids [71,72]. By analyzing the 3D structures of actinoporins, it has been shown that the β-sandwich structure remains intact upon oligomerization [56,69].

The pore stoichiometry and its composition are not clear yet. Several analyses have demonstrated that the stoichiometry of the pores formed by actinoporins is heterogeneous. For StnI, StnII, and EqtII a model was proposed where three and four monomers participate together with lipids to allow for pore formation [69,70,71,72]. In a recent report, by site-directed spin labeling and electron paramagnetic resonance spectroscopy, the pore formed by StnI was shown to have an architecture of eight monomers [73]. The FraC pore was established by nine or eight protomers with the hydrophobic face positioned outwards from the oligomer and hydrophilic face toward the pore light. This pore model is known as an α-helical bundle and only involves protein-protein interactions [58]. However, subsequent work with FraC was based on thermodynamic, functional, and structural analysis of the hybrid protein/lipid pore. This work describes the route of activation of the monomer until its insertion in the membrane and pore formation by eight monomers (Figure 1C) [42,74]. Although the mechanism of action of actinoporins is not yet fully known, their oligomerization has impacted some biotechnological areas, and they have been considered as candidates for the design of molecular tools generated by protein engineering [47,75,76,77].

## 3. Therapeutic and Biotechnological Tools Based on Actinoporins

### 3.1. Actinoporin-Based Immunotoxin For Cancer Therapy

Due to its high morbidity and mortality, cancer has emerged as one of the leading world public health problems worldwide [80]. It has been estimated that the cost resulting from the increase of new cancer cases will grow to more than twenty million dollars annually by 2025 [81,82]. Therapeutic methods such as radiotherapy, surgery, and chemotherapy for the treatment of cancer still exhibit limited success, as only a small improvement has been observed in mortality rates from common cancers, and the risk of recurrence remains high [83,84]. Collateral damage to healthy cells is a problem encountered when using radiation and chemotherapy, and these treatments are inefficient against solid tumors due to their inability to penetrate the tumor mass [85,86]. Research studies are currently examining the design of new drugs that possess a higher therapeutic index based on improvements to their specificity and selectivity towards cancer cells. The goal of these new therapeutic design strategies is to more effectively target tumor cells while simultaneously reducing the harmful side effects of these therapies on healthy tissues [87,88,89].

Many tumor cells exhibit changes in the lipid composition of their membranes in comparison to healthy tissue. These changes specifically include increased synthesis of SM and Chol [26,27]. Actinoporins have been assayed in synergy with anticancer drugs and found to increase their efficacy of these drugs. Although the cytolytic activity of APs is not specific to a cell type, applications are mainly focused in the biomedicine area because of their ability to produce lysis in cancer cells through pores. To enhance cellular target specificity APs has been integrated into an immunotoxin design. This application of the APs could avoid internalization of the toxin into the cell and prevent collateral damage commonly caused by chemotherapy [28].

Protein engineering has led to the design of immunotoxins that can be fused to various antibodies [6,7,8]. Some actinoporins such as EqtII, FraC, StnI, and Gigantoxin-4 have been used as a toxic fraction in the design of immunotoxins [4,11,30,31,32,90]. EqtII was chemically coupled with transferrin (Tf) by disulfide bond formation using N-succinimidyl 3-(2-pyridyl-dithio) propionate (SPDP) to direct its cytotoxic activity toward cancer cells in vitro. However, the conjugate demonstrated nonspecific activity, and it was attributed to a residual ability of the toxic fraction to bind to membrane lipids [89]. The mechanism of action underlying the function of this hybrid was not entirely clear, but the design was based on an activation mechanism that was induced by the acidic pH found in the environment of the endocytic compartment. This pH would allow for the separation of the two components by reducing the disulfide bond and thus freeing EqtII. In other reports, three mutants of EqtII (K20C, R126C, and A179C) were selected from a panel of twenty mutants, and the chosen mutants could be biotinylated without altering their cytotoxic activity [88]. This system consisted of a primary antibody that recognized a membrane antigen of the human malignant melanoma A375 cell line and a secondary antibody that recognized the primary antibody and was coupled to biotin, ultimately allowing for avidin binding to the biotinylated mutants of EqtII. This molecular complex specifically directed the cytotoxic activity of these EqtII mutants to be specifically directed towards A374 cells [88]. In another scenario, StnI was coupled to the murine monoclonal antibody IOR-C5 that recognizes a specific tumor antigen (IOR-C2). The coupling was performed using sulfosuccinimidyl 4-(N-maleimidomethyl)-cyclohexane-1-carboxylate (SMCC) to obtain two-hybrid molecules that were composed of the antibody and one or two StnI molecules [30]. The hybrid possessing two toxin molecules was the only one capable of effectively eliminating SW948 colon cancer cells. However, the immunotoxin maintained hemolytic activity [30]. Gigantoxin-4 is an actinoporin isolated from *S. gigantea* and is fused with the 4D5 scFv antibody that is part of an immunotoxin called Gigantoxin-4-4D5 scFv. This conjugate was used to target SK-OV-3 human ovarian cancer cells and possessed higher cytotoxic activity compared to that of Gigantoxin-4 [32].

Proteases have been exploited in the mechanism of action of immunotoxin. Cathepsins, kallikreins, serine proteases, and matrix metalloproteinases (MMPs) are highly expressed in cancer tumors intracellularly or extracellularly [90,91,92,93]. EqtII protoxin was designed based on the I18C mutation (EqtII-I18C), and this variant was coupled to a biotinylated peptide (NH2-CNKSRLGLGK-biotin-COOH) that allowed for the attachment of avidin to obtain the EqtII-I18C-S-S-peptide-biotin-avidin complex [31]. This biotinylated peptide can be cleaved by both cathepsin B and matrix metalloproteases (MMPs). The hemolytic activity of the complex in human erythrocytes was considerably suppressed. However, treatment of the complex with dithiothreitol (DTT) restored the hemolytic capacity of EqtII-I18C. The cytotoxic activity of the complex was evaluated in MCF7 and ZR751 breast adenocarcinoma cells and human fibrosarcoma cells (HT1080) [31]. The complex exhibited differential activities in cancer cell lines and erythrocytes, indicating that activation of the conjugate was achieved through the enzymatic activity of cathepsin B and MMP [31].

The generation of immunotoxins was recently reported whereby FraC was used as the toxin fraction [4]. Through a strategy of directed evolution, it was possible to obtain an immunotoxin composed of three elements. The first element is a camelid nanoantibody (Nb) that recognizes the epidermal growth factor receptor (EGFR), an epitope present in several types of solid tumors. Thus, the molecule can be directed to a specific target and covalently linked to the second component, which is the toxic effector that bound through an amino acid linker peptide (18 amino acid residues) to the toxic fraction that is represented by FraC. The last element is an activator composed of a hydrophilic protein (dihydrofolate reductase, DHFR) covalently coupled to the N-terminal region of FraC through an 18 amino acid linker peptide that contains the cleavage site for the protease furin. DHFR functions as a blocker of the transmembrane site of the toxic fraction to allow the targeting element (nanoantibody) and the protein activator (furin cutting site) to regulate toxin activity. This immunotoxin has no activity in cancer cells but is proteolyzed by protease cancer cells [4].

The immunotoxin size and the affinity of binding to the molecular target affect the penetration and diffusion within the tumor tissue. The differences in lytic potential and stoichiometry of the pores formed by APs could be attributed to the differences in the primary structure, particularly in the N-terminal [60]. It has been observed that the N-terminal of actinoporins may be sufficient to destabilize the cell membrane and cause lysis [94,95]. Therefore, the design of immunotoxins that possess much smaller molecular weights could be feasible, if minimal regions, that conserve toxic antibody activity and improved affinity could eventually improve molecular diffusion and decrease the immunogenicity of these molecules.

A new alternative to immunotoxin has been developed based on a mutant PFT combined with a photo-activated molecular switch which control oligomeric pore formation [96]. FraC mutants (K77C and W112C) were produced where residues in crucial positions were required for the lipid protein or protein-protein interaction. Other mutated residues included G145, N147, and S166, which are located in one of the loops in the sphingomyelin protomer interface; G13, which is found in the α-helix that is part of the transmembrane pore; and Q130, E134, and Y138, which are located in the α-helix 2, where their side chains are on the membrane surface. The individual Cys mutants are then conjugate to an azobenzene molecule that functions as a switch that regulates PFT activity by changing from its trans (inactive) to cis (active) configurations when the molecule is irradiated at 364 nm. The authors demonstrated that the design could be photo-controlled by irradiating the protein in its cis states with white light to interrupt the formation of pores. This design applies not only to phototherapy in cancer but also to allow for the more precise formation of nanopore matrices for stochastic analysis, which is discussed in more detail below [96].

**Figure 2:**
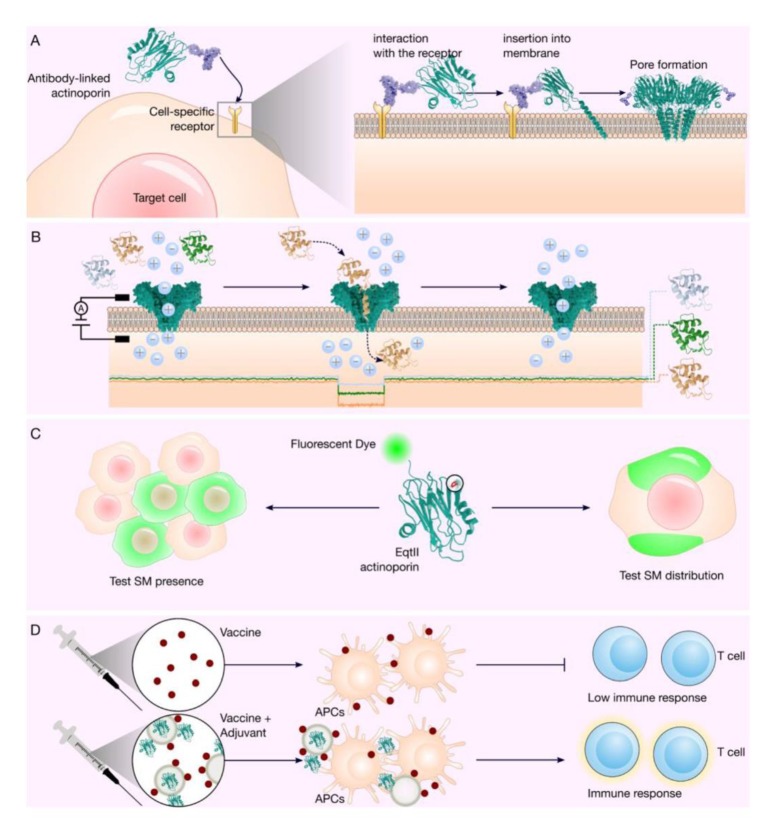
Actinoporin applications. (**A**). Actinoporin-based immunotoxins act at the membrane level forming a pore. The immunotoxin recognizes a specific receptor in the target cell, after the toxin binds to the plasma membrane, subsequently acquiring a conformational change for the α-helix of the N-terminal region to internalize in the membrane, concluding with the formation of a pore, where finally, an osmotic imbalance and finally cell death could occur in the cell [46,148]. (**B**). Stochastic biosensors based on actinoporins. From a mixture of polypeptides, a unique polypeptide can be differentiated from ionic current blockages; this characteristic has considered as a fingerprint registered in a known database of these types of records [39]. (**C**). By protein engineering, an Ap-GFP chimera can be obtained, where the AP is a mutant without hemolytic activity but maintains the affinity to SM, which allows its application for the study of the distribution of SM in cell membranes [140]. (**D**). Actinoporins as adjuvants is a novel application of these proteins in vaccine development. Encapsulated actinoporin (APC) in a lipid formulation (gray circles), can induce a potent Ag-specific CTL response compared to only the vaccine (brown dots). It is important to note that the property of the AP (green monomeric structure) as an adjuvant is independent of its pore formation capacity, having been tested with a mutant of StnII (monomeric protein structure in green) lacking this function. In the formulation, it was found that non-encapsulated AP could induce DC maturation and contribute to the encapsulated St-mediated immune response [43].

Immunotoxins based on actinoporins are ideal candidates for application as immunotherapy in solid tumors because their effect is at the membrane level, and they do not need to be internalized in the cell [11] (Figure 2A). Regarding immunoreaction, the epitope mapping and mutation strategy could be successful with actinoporins, as has been achieved with the immunotoxin moxetumomab pasudotox-tdfk (LUMOXITI^®^, AstraZeneca Pharmaceuticals LP), which has a truncated protein toxin of *Pseudomonas* exotoxin [9,97,98]. More studies examining the structure function relationship and stability of possible actinoporin variants will be essential for the development of therapeutic proteins based on this type of PFT.

### 3.2. Biosensors Based On Actinoporins Nanopores

PFTs are considered an important biotechnological tool to design biosensors based on protein nanopores, and these have been used to detect different analytes, including nucleic acids and polypeptides [99,100,101]. Stochastic biosensors detect individual molecules based on the alteration or partial blockage of the electrical current generated by the molecules as they pass through the nanopore [101,102]. The nanopores are embedded in membranes with two compartments (cis and trans) that contain a solution of electrolytes and electrodes. When a polarization voltage is applied across the membrane, the free passage of ions through the nanopores generates a constant current that is indicative of an open pore [103,104]. When a molecule diffuses through the pore, the ion flow is partially interrupted and results in a change in the ionic current that is detected as an electrical signal [105,106]. This technique is called resistive pulse, and it allows researchers to detect and identify an individual molecule from its volume, concentration, and pore interaction. During the process, the resistive current pulse, the frequency of pore block events, and the time the analyte takes to pass through the pore detection zone are measured. These parameters allow a molecule to be identified according to its distinctive register that is based on its capacity to block the pore [106,107,108].

The development of sensors based on protein nanopores has led to significant advances in the analysis of nucleic acids (DNA and RNA) [109,110,111]. Oxford Nanopore Technologies (ONT) was the first to launch a DNA sequencer based on protein nanopores constructed from a mutant of the Curli production assembly/transport component (CsgG) protein from *Escherichia coli* [112,113]. This portable device is known as MinION and has been used in the analysis of nucleic acids to determine the small metagenomics of bacterial communities and to sequence viral genomes and genes from human cell lines [114,115,116,117]. Despite still possessing a higher error rate compared to that of other sequencing technologies, MinION is advantageous in that it can analyze data in real-time and in situ, ultimately making this type of technology ideal for use in the field and hospital diagnoses for the identification of pathogens [113,118,119]. In addition to nucleic acid sequencing, other areas of analysis are focused on the design of sensors based on protein nanopores, such as the identification of peptides and proteins, the monitoring of enzymatic reactions, protein folding, and glucose detection [120,121,122].

Unlike polynucleotides, polypeptides do not possess a homogeneous charge, because the side chains possess amino acids with positive or negative charges. Some of the factors that must be considered when using nanopores for the recognition of peptides and proteins are the shape, size, surface, and internal charge of the pore [40]. In the case of nucleic acids, an electric field is used to control and stretch the molecules that translocate through the protein nanopore. However, the polypeptides do not exhibit homogeneous charge, and therefore, their entrance to the nanopore depends upon the electroosmotic flow (EOF), which is the directional flow of water through the nanopore [123,124]. The EOF functions as the driving force that transports the polypeptides through the pore and is induced by the fixed charge of the inner walls of the nanopore [123,124,125].

The analyte speed to cross and the selectivity of the nanopore are the main problems in the single-molecule study. Therefore, nanopores possessing characteristics that can determine the size and hydrophilic and hydrophobic properties are required to increase the interaction or delay the analyte displacement time inside the pore [120,126,127]. Pore forming proteins have been chemically modified to change the size of the pores, modify the load distributions, improve sensitivity and stability, and to expand the detection capacity in a more specific manner. The protein forming pores most frequently used in the design of nanopores include α-HL [122,128], aerolysin [128], *Mycobacterium smegmatis* porin A (MspA) [129,130], CsgG from *E. coli* [131], and FraC from the sea anemone *A. fragacea* [39,42].

The pores designed using FraC exhibit remarkable characteristics that make them candidates for the design of nanopores that can be applied to DNA analysis [77]. The pore design using the wild type of FraC contains eight subunits, and the three-dimensional structure of the pore has been determined [74]. FraC protein has been modified to increase the efficiency of DNA analysis to distinguish ssDNA homopolymers, and by changing only one amino acid residue, the effectiveness in the formation of uniform pores was increased [42,77]. FraC possesses the plasticity to design functional pores with adjustable diameters to monitor peptides [42]. The pores of the recombinant FraC have been used at different potentials applied in the trans electrode and at two pH conditions (pH 7.5 and 4.5) [42] to assess their ability to identify a mixture of polypeptides with different molecular weights [39,132]. FraC nanopores could even discern between two peptides, each one with a single modification in one residue based on current blockages, and they could also differentiate between two protein populations at different concentrations [132]. Through protein engineering in FraC, electro-osmotic flow can be generated in a specific direction to identify polypeptides by size [39]. Based on this, it has been proposed that FraC pores possess an excellent potential for use in the development of possible peptide sequencing methods based on protein nanopores (Figure 2B). Several strategies are being evaluated using other PFTs to analyze and sequence peptides with greater robustness. These strategies include the coupling of enzymatic activities such as proteases or the insertion into the pore of ligand molecules that allow the problem analyte to be recognized with high sensitivity [37].

### 3.3. Actinoporins Used to Identify SM in the Cell Membranes

The specific recognition of SM by actinoporins has led to these proteins have been used as a molecular probe that allows for the identification and analysis of the distribution of this lipid in eukaryotic cells [24,25,133]. SM is predominantly found in the outer face of the plasma membrane of vertebrate cells, where it forms lipid microdomains in combination with cholesterol that are termed lipid rafts that function as sites of cellular entry or exit [134,135]. SM also is a precursor for bioactive lipids such as ceramide, ceramide-1-phosphate, sphingosine, and sphingosine-1-phosphate, and these lipids participate in the regulation of cell proliferation, differentiation, and apoptosis [136,137].

StnII has been used in probes to recognize SM in cells. This actinoporin was purified from its natural source using chromatographic methods to produce IgG monoclonal antibodies by immunizing BALB/c mice. The distribution of SM in neuroepithelioma cell lines (HCP-100) and human dermal fibroblasts (HDFs) was evaluated by fluorescence microscopy using subtoxic StII concentrations in combination with primary (A10) and secondary (Texas Red-conjugated) antibody systems. The sequential addition of StII, A10, and Texas Red-conjugated goat anti-mouse revealed that StII is capable of binding to the cell surface of both human cell lines. However, pretreatment with sphingomyelinase significantly reduced the fluorescence signal, which means that the signal derives from the interaction between the SM and the StII [24]. The StII/A10 system has been used to evaluate the presence and distribution of Chol-SM concerning Chol-GM1 in cells obtained from patients diagnosed with Niemann Pick C disease (NPC), a lysosomal disease characterized by the deposition of intracellular lipids in different tissues [24]. The StII/A10 system was able to recognize a class well defined as lipid storage vesicles in fibroblasts from persons with NPC. Therefore, StII can potentially be used in the design of molecular probes for the detection of soluble and membrane-coupled SM [24].

EqtII has also been used for the design of fluorescent probes. The EqtII gene was fused to that of the green fluorescent protein (GFP) and was expressed in *E. coli*. EqtII-GFP exhibited decreased hemolytic activity compared to that of EqtII wt. However, its ability to bind to SM was not altered [138]. The EqtII-GFP chimera was expressed in the MDCK-II canine cell line to explore the distribution of SM. EqtII-GFP located on the apical side of the plasma membrane, indicating how SM are distributed in the polarized membranes of MDCK-II cells [138].

Point mutations in the EqtII have shown which amino acid residues are important for the recognition of SM as well as for hemolytic activity. It has shown that amino acid residues W112 and Y113 are essential for the recognition of SM, and mutation by alanine decreases the binding of the toxin to SM [139]. Based on these observations, these mutants were fused with GFP and expressed in MDCK-II cells, showing an inability to bind to the plasma membrane. On the other hand, the EqtII-V8C/K69C mutant expressed in MDCK-II cells maintained the ability to bind to SM; however, it lost its hemolytic activity [140], because the formation of a disulfide bond between the residues C8 and C69 immobilizes the N-terminal connected to the core of the β-sandwich, and its translocation to the membrane necessary for the formation of pores is prevented [140], however, under reducing conditions the protein recovers its hemolytic activity [140].

EqtII from sea anemone *A. equina* conjugated to GFP revealed the ability of these toxins to recognize SM in different location on the cell membranes [25,140], in that lysenin recognized the grouped SM, while EqtII distinguished the dispersed SM. There are currently a limited number of proteins that possess the ability to specifically recognize membrane lipids, such as SM [141,142]. This ability of actinoporins is a characteristic that makes these molecules ideal candidates for potential application in the design of tools aimed at analyzing the distribution and dynamics of membrane lipids (Figure 2C). Although the intrinsic toxicity of these molecules is a limitation, this could be addressed by generating mutants with depleted cytotoxic and hemolytic activity.

### 3.4. Actinoporins As an Adjuvant for Vaccine Design

One challenge in the development of vaccines is to improve the immune response mediated by cytotoxic T lymphocyte (CTL) CD8^+^. It is necessary that the exogenous antigens must overcome the plasma membrane barrier and enter the cytosol of the antigen-presenting cells (APCs), where the immune cells are capable of processing these molecules to inhibit the antigen recognition by lymphocytes [143,144]. Liposomes, such as those used to target T cells, have shown potential use for immunization and vaccine design due to their ability to transfer and release antigenic molecules in APCs and to stimulate the immune response [145,146,147]. It has been previously reported that StII and StII, toxins from the sea anemone *S. helianthus* encapsulated in a LP/OVA/StII formulation (mixture of SM-free lipid, ovalbumin and the toxin StII) were used to assess their stimulatory effect on a population of Ag-specific CTLs [43]. This suggests that LP/OVA/StII promotes the expansion of antigen-specific CD8+ T cells compared to that achieved using the liposome formulation that contains only the model antigen (LP/OVA). It has also been shown that LP/OVA/ StII is capable of inducing lysis in target cells in the absence of CD4+ T cells, ultimately promoting the specific antigen response by CD8+ CTLs. An StI W111C mutant was obtained, which had no cytolytic activity and was encapsulated like StII, showing the ability to induce Ag-specific CTL response comparable to the formulation with StII. This report has shown that actinoporins can function as immunomodulators in a liposome system by improving the specific cytotoxic immune response of antigen in vivo and can even improve the antitumor response in a preventive scenario (Figure 2D) [43].

## 4. Conclusions

Sea anemones have become a rich source of molecules with high potential in biomedicine and biotechnology [12]. Actinoporins are produced by sea anemones and are considered as effective pore-forming toxins due to their plasticity, which enables them to change their conformation from the monomeric to the oligomeric state [56,68], and their high affinity for sphingomyelin [24,61], the principal characteristics of these proteins and the main principle of their applications.

The structural characterization of APs focused in the monomeric, and the oligomeric state to form a pore has allowed a substantial advance in the application of these biomolecules, as is the case of EqtII, StnI and II, and FraC. EqtII engineering has made it possible to elucidate the process of the formation of the oligomer in membranes [66,76,149,150,151]. The FraC structure allows corroborating the AP regions to have the most significant conformational change, which consists mainly of the N-terminal α-helix, as well as the region of the β-sheets that maintain their structure in the formation of the pore in the membrane [58]. The ability that a PFT can acquire when fused with an antibody has allowed the design of immunotoxins [91], which by protein engineering, can change their affinity to different cells. However, it was reported that an AP from *Heteractis crispa* (*Radianthus macrodactylus*), may have two properties, namely cancer-preventive and anticancer cytotoxic, without modifying its primary sequence [152], which demonstrates the high capacity in cancer therapy.

The development of biosensors, mainly in conjunction with bacterial PFTs, has demonstrated the scope of its application for the sequencing of small metagenomes, or analysis of unique polypeptide molecules. With regard to significant incorporation of FraC into nanopores design, however work is still being done to make the biosensor more efficient with this actinoporin.

Two novel applications have been reported for APs, as adjuvants in the design of vaccines to increase the response CTL [43], and as a sensor in the identification and dynamics of SM in cell membranes [24], although for the application in vaccine design, adjuvant molecular. The high affinity that APs have for SM is a very striking tool, considering that SM predominates in the vertebrate cell membranes, and can function as a receptor for bacteria toxin [153] and viral proteins such as the Ebola virus that predominantly joins SM-rich regions [154]. It can also be used in apoptosis process analysis [155] well as the study of the processes of atherosclerosis [156] among others.

Therefore, knowledge of APs should be extended to diverse areas. Actinoporins will likely continue to surprise many in the various fields of research in which they can be applied, while at the same time, contribute to the basic knowledge of pore-forming toxins.

## Abbreviations

Pore forming toxins (PFTs), actinoporin (AP), sphingomyelin (SM), phosphocholine (POC), phosphatidylcholine (PC), cholesterol (Chol), equinatoxin (Eqt), sticholysin (St or Stn), fragaceatoxin (FraC).
